# Nitrate-Rich Beetroot Juice Reduces Blood Pressure in Tanzanian Adults with Elevated Blood Pressure: A Double-Blind Randomized Controlled Feasibility Trial

**DOI:** 10.1093/jn/nxaa170

**Published:** 2020-07-29

**Authors:** Mario Siervo, Oliver Shannon, Navneet Kandhari, Meghna Prabhakar, William Fostier, Christina Köchl, Jane Rogathi, Gloria Temu, Blossom C M Stephan, William K Gray, Irene Haule, Stella-Maria Paddick, Blandina T Mmbaga, Richard Walker

**Affiliations:** School of Life Sciences, University of Nottingham, Nottingham, United Kingdom; Human Nutrition Research Centre, Population Health Sciences Institute, Newcastle University, Newcastle on Tyne, United Kingdom; Human Nutrition Research Centre, Population Health Sciences Institute, Newcastle University, Newcastle on Tyne, United Kingdom; Faculty of Medical Sciences, Newcastle University, Newcastle on Tyne, United Kingdom; Faculty of Medical Sciences, Newcastle University, Newcastle on Tyne, United Kingdom; Human Nutrition Research Centre, Population Health Sciences Institute, Newcastle University, Newcastle on Tyne, United Kingdom; Human Nutrition Research Centre, Population Health Sciences Institute, Newcastle University, Newcastle on Tyne, United Kingdom; Kilimanjaro Christian Medical University College, Moshi, Tanzania; Kilimanjaro Christian Medical University College, Moshi, Tanzania; Institute of Mental Health, University of Nottingham, Nottingham, United Kingdom; Northumbria Healthcare NHS Foundation Trust, North Shields, United Kingdom; District Medical Officer, Hai District Hospital, Bomangombe, Tanzania; Clinical and Translational Medicine, Newcastle University, Newcastle on Tyne, United Kingdom; Kilimanjaro Christian Medical University College, Moshi, Tanzania; Northumbria Healthcare NHS Foundation Trust, North Shields, United Kingdom; Population of Health Sciences Institute, Newcastle University, Newcastle on Tyne, United Kingdom

**Keywords:** dietary nitrate, folate supplementation, hypertension, Tanzania, blood pressure

## Abstract

**Background:**

In Sub-Saharan Africa, current strategies are struggling to control the burgeoning hypertension epidemic. Dietary interventions such as inorganic nitrate or folic acid supplementation could represent promising strategies for reducing blood pressure (BP) in this setting.

**Objectives:**

This feasibility study explores the effects of dietary inorganic nitrate supplementation, alone or in combination with folic acid, on BP in Tanzanian adults with elevated BP in Tanzania.

**Methods:**

A placebo-controlled, double-blind, randomized controlled feasibility trial was conducted. Forty-seven middle-aged and older participants (age: 50–70 y, BMI: 26.3–29.1 kg/m^2^) were randomly assigned to 3 conditions for a period of 60 d: *1*) high-nitrate beetroot juice (∼400 mg nitrate) and folic acid (∼5 mg folic acid) (N + F), *2*) high-nitrate beetroot juice and placebo (N + P), or *3*) nitrate-depleted beetroot juice and placebo (P + P). Clinic and 24-h ambulatory BP and measurements of compliance in plasma (nitrate and folate concentrations) and saliva (nitrate and nitrite) were obtained at baseline, 30 d, and 60 d.

**Results:**

Baseline resting systolic and diastolic BP (mean ± SD) was 151.0 ± 19.4 mm Hg and 91.8 ± 11.7 mm Hg, respectively. Compliance to the interventions was high (>90%) in all groups which was confirmed by the significant increase in nitrate and folic acid concentrations in plasma and saliva samples in the treatment arms. After 60 d, 24-h systolic BP dropped by −10.8 ± 9.8 mm Hg (*P* < 0.001), −6.1 ± 13.2 mm Hg (*P* = 0.03), and −0.3 ± 9.7 mm Hg (*P* = 0.83) in the N + P, N + F, and P + P groups, respectively. There was a significant decrease in 24-h diastolic BP in the N + P group (−5.4 ± 5.0 mm Hg, *P* = 0.004), whereas changes were not significant in the N + F (−1.8 ± 8.1 mm Hg, *P* = 0.32) and P + P (1.6 ± 8.3 mm Hg, *P* = 0.43) groups.

**Conclusions:**

Dietary inorganic nitrate represents a potential nutritional strategy to lessen the hypertension epidemic in Sub-Saharan Africa. These findings support the rationale for future long-term investigations exploring the efficacy of dietary nitrate for lowering BP and attenuating cardiovascular disease risk in this setting.

This trial was registered at isrctn.com as ISRCTN67978523.

See corresponding commentary on page 2233.

## Introduction

Hypertension is associated with an increased risk of stroke, ischemic heart disease, chronic kidney disease, and neurodegeneration, and is a major contributor toward disability and premature mortality (1–3). In 2010, 28.5% of adults living in high-income countries and 31.5% in low- and middle-income countries (LMICs) had hypertension worldwide (3). Almost two-thirds of all hypertensive individuals live in LMICs, and the highest rates of hypertension are found in Africa where 46% of adults aged ≥25 y have raised blood pressure (BP) (4). In Sub-Saharan Africa (5), this condition was the sixth leading cause of disability, accounting for >11 million disability-adjusted life years, in 2010 (6), and was estimated to have caused over half a million deaths and 10 million years of life lost in 2010 (1, 7).

The United Republic of Tanzania is a low-income country, where current management strategies are struggling to control the burgeoning hypertension epidemic. Indeed, a study from our group conducted in Tanzania found that only one-third of hypertensive individuals had previously been diagnosed, only one-sixth of those diagnosed were on antihypertension treatment, and only one-sixth of those being treated exhibited adequate BP control (8). These findings highlight the need for improved diagnosis of hypertension in Tanzania. They also demonstrate the crucial need to explore alternative ways of reducing BP in this setting.

Dietary interventions could play an important role in this regard. Firstly, dietary interventions may help by reducing the strain on resource-limited health care systems, where the availability and affordability of medication are often restrictive. Secondly, individuals may be more receptive to consuming bioactive foods or nutritional supplements, rather than antihypertensive medications, owing to the reduced risk of side effects and negative cultural perceptions of Western medicine (9). Two dietary interventions that offer promise in this regard are inorganic nitrate and folic acid.

Dietary inorganic nitrate, which serves as a substrate for the pleiotropic gasotransmitter nitric oxide (NO), is found in large quantities in green leafy vegetables and beetroot (10), and has attracted considerable interest in the past decade as a nutritional antihypertensive agent (11–14). In their seminal study, Larsen et al. (11) reported a 3.7 mm Hg decrease in diastolic BP after 3 d supplementation with sodium nitrate. Subsequently, Webb et al. (12) reported a ∼10.4 mm Hg and ∼8.0 mm Hg reduction in systolic and diastolic BP, respectively, 2.5–3 h after acute ingestion of nitrate-rich beetroot juice. Similar benefits have also been reported in a group of African-American women (15). A recent meta-analysis reported overall reductions in systolic and diastolic BP of 4.8 mm Hg and 1.7 mm Hg, respectively, after dietary nitrate supplementation (13). Folic acid has also attracted attention as a potential BP-reducing nutritional compound. It has been demonstrated to improve endothelial function and reduce BP: effects which may be underpinned by improved NO synthase coupling, scavenging of superoxide, and a reduction in homocysteine (16–18). Given that dietary nitrate and folic acid operate, at least in part, through separate mechanisms, it is possible that they may elicit additive or synergistic effects on BP. If shown to be effective, the consumption of nitrate- and folic acid–rich foods could be encouraged to help combat raised BP levels in Tanzania, with potential applications in other African countries. However, there are presently no data on the combined effects of consuming these 2 nutritional supplements, nor their efficacy in this setting.

The objective of this feasibility study was to obtain information on the effects of dietary inorganic nitrate supplementation, alone or in combination with folic acid supplementation, on BP in a group of adult individuals with elevated BP living in the United Republic of Tanzania. Specifically, we utilized resting clinic blood pressure measurements (CBPMs) and ambulatory 24-h blood pressure monitoring (ABPM) to examine changes in systolic and diastolic BP. Blood and saliva samples were collected to evaluate compliance to the interventions (folic acid and nitrate) and measure biomarkers of NO production.

## Methods

### Ethics and consent

The study (ISRCTN67978523) was approved by the Newcastle University (1458/3377/2018), Kilimanjaro Christian Medical Centre (No. 2258), and National Institute of Medical Research in Tanzania (NIMR/HQ/R.8a/Vol. IX/2738) ethics committees. Participants initially gave their verbal consent during the recruitment phase and written informed consent was subsequently given by eligible participants before entering the study. If participants were unable to sign, thumbprints were obtained before enrolment to acknowledge their consent to participate.

### Study design

We conducted a 3-arm, placebo-controlled, double-blind, randomized controlled feasibility trial in the Hai Demographic Surveillance Site in the Kilimanjaro region of Northern Tanzania. Participants attended during their visits the Hai District Hospital and Kware village dispensary and biological samples were processed and stored at the Kilimanjaro Clinical Research Institute (KCRI). More detailed information on the study protocol has been previously described (19). The trial is characterized by 4 stages: *1*) recruitment, *2*) screening, *3*) randomization and baseline assessment, and *4*) intervention and end of study assessment. Eligible participants were randomly allocated to 1 of the 60-d interventions:

- Group 1: combined intervention (high-nitrate beetroot juice and folic acid, N + F)
- Group 2: single intervention (high-nitrate beetroot juice and placebo, N + P)
- Group 3: control (nitrate-depleted beetroot juice and placebo, P + P).

### Recruitment

Village representatives who had previously helped in conducting other research projects in the same region were trained and assisted with the identification of eligible participants in their villages. At the home visit, representatives recorded basic contact details and measured resting BP and eligible participants were invited to a full screening assessment at 1 of the 2 research centers (Hai District Hospital or Kware village dispensary).

### Screening

The screening phase included 2 assessment visits (A and B). At visit A, information concerning drugs, diet, and smoking; anthropometrics (weight, height, waist circumference); and resting CBPMs were collected. Eligible participants were then invited to attend screening visit B to collect additional information on dietary and lifestyle habits, knowledge of hypertension, and dietary interventions used in the trial.

### Randomization and baseline

Block-randomization was utilized to generate a random sequence of codes for each intervention and participants were assigned into 1 of the 3 arms as they passed the eligibility assessment at the screening visits. Participants and the research team were blinded to the interventions and each group received the nutritional supplements at the baseline visits.

### Primary and secondary outcomes

The primary outcome of the trial was to evaluate feasibility, acceptability, and compliance to the interventions and to the study protocols. Secondary outcomes included changes in 24-h ambulatory and resting clinic BP; plasma homocysteine, C-reactive protein (CRP), folate, and nitrate; and salivary nitrate and nitrite concentrations.

### Participants

Participants were eligible if their age ranged between 50 and 70 y, they were nonsmokers, had a mean systolic BP between 130 and 170 mm Hg, and a BMI (in kg/m^2^) ranging from 18.0 to 40.0. Exclusion criteria were determined by factors likely to affect the study outcomes owing to their influence on cardiometabolic function, vascular function, and participant compliance. **Supplemental Table 1** provides a list of all the inclusion and exclusion criteria. Those who were excluded were given appropriate medical advice and referred to their local health services for follow-up.

### Intervention

After the randomization and baseline measures, 16 participants were assigned to each one of the 3 intervention groups. Measurements were taken at the research sites at baseline (day 0), interim (day 30), and at the end of the study (day 60). A telephone interview was conducted ∼15 d after commencing the intervention to assess participant adherence and any health and safety concerns. Participants were asked to maintain their usual dietary habits, physical activity level, and consumption of alcohol and caffeinated drinks during the trial. They were also asked to avoid using mouthwash during the study period, given that this is known to diminish the physiological effects of dietary nitrate (20). All capsules (used for administering folate and folate placebos) were stored in identical white containers showing the individual participant code, the number of capsules, the expiry date, and storage and prescription instructions. Subjects were provided with a form to record the time of supplement consumption and any problems they had with the interventions. The intervention groups were as follows.

#### N + F: “combined intervention: nitrate-rich beetroot juice and folate capsule”

Participants were given 1 bottle (70 mL) of concentrated beetroot juice (Beet It shots, James White Ltd) and 1 capsule of folate to be taken every morning. The juice corresponded to a supplementation of ∼400 mg inorganic nitrate per day, and each capsule contained 5 mg folate (folic acid, 5 mg, Bio-Tech Pharmacal Inc.).

#### N + P: “single intervention: nitrate-rich beetroot juice and placebo capsule”

Participants were given the same bottle (70 mL) of concentrated beetroot juice as group 1 (Beet It, James White Ltd) and 1 placebo capsule containing sucrose powder to be taken every morning.

#### P + P: “control group: placebo juice and placebo capsule”

Participants were given 1 bottle (70 mL) of nitrate-depleted beetroot juice (<1 mg inorganic nitrate, James White Ltd) and a placebo capsule containing sucrose powder to be taken every morning. This juice had the same appearance, color, and taste as the nitrate-rich beetroot juice, and the placebo capsules had the same color and appearance as the folic acid capsules.

### Study measures

#### CBPMs

Resting CBPMs were carried out using a calibrated automatic device (Omron M3, OMRON HealthCARE UK, Milton Keynes, UK). Participants were asked to rest in a sitting position for ≥15 min, and BP was measured on the left upper arm supported at the level of the heart. Three measurements were taken with a 1-min break in between each measurement. The mean of the second and third values was recorded and used in the study.

#### ABPM

Participants were fitted with an ABPM system (Mobil‐O‐Graph, Stolberg, Germany) consisting of an inflatable cuff attached to a small monitoring device. The cuff was secured around the individual's left upper arm. Readings were then taken every 30 min in the day from 08:00 to 20:00 h (day time) and every hour overnight from 20:00 to 08:00 h the subsequent day (night time). Analyses were conducted for the entire 24-h recording period (24-h) and separately for the day and night recording periods.

#### Salivary strips

Salivary nitrite was measured using commercially available test strips (Berkeley test strips) at the study visits to evaluate their validity as a measure of longer-term compliance to nitrate interventions. A strip was placed on the tongue which changes color upon saliva exposure. The participant's salivary nitrite concentrations were graded against a color wheel supplied by the manufacturer.

#### Blood samples

At screening A, all participants had their full blood count measured for hemoglobin concentrations to screen for anemia. For eligible participants, blood samples were collected in 9-mL lithium heparinized tubes at each study visit. After collection, blood samples were immediately labeled and kept in ice boxes before being transferred to the KCRI, where they were centrifuged at 4°C, 4000 × *g* for 10 min and divided into 3 plasma aliquots and stored at −80°C until shipment to the United Kingdom. Plasma concentrations of folic acid (MET-5068, Cell Biolabs), CRP (KHA0031, Invitrogen, Thermo Fisher Scientific Inc.), nitro-tyrosine (ab210603, Abcam), and homocysteine (STA-670, Cell Biolabs) were determined in duplicate using quantitative ELISA kits according to the manufacturer's instructions. Nitrate concentrations in plasma and saliva were measured by ozone-based chemiluminescence (Sievers NOA 280i, Analytix). The **Supplemental Methods** provide a brief description of the chemiluminescence analysis.

### Sample size

A formal statistical calculation of the sample size was not possible owing to the lack of data on the effects of dietary nitrate on cardiometabolic outcomes in sub-Saharan African populations. Therefore, we calculated our sample size using the approach for pilot trials proposed by Whitehead et al. (21). A sample size of 15 participants/arm would allow the detection of a medium effect size (δ = 0.30–0.70) with a power of 0.80 and a *P* value < 0.05. In addition, we also used data from a published trial testing the effects of dietary nitrate on BP in hypertensive British patients to further support the appropriateness of the sample size calculation (22). The trial reported a significant effect of inorganic nitrate supplementation on systolic 24-h ABPM after 6 wk of supplementation in 68 drug-naïve hypertensive patients. The difference in mean systolic BP between the nitrate and placebo groups was −7.7 mm Hg (95% CI: 4.1, 11.2 mm Hg) with an effect size of 0.39. Applying an ANOVA model for repeated measures, we estimated that 14 participants/group (total sample size: 42) would be sufficient to detect a significant difference between the placebo and the nitrate intervention group with a *P* level of 0.05 and 80% power. Our study aimed to recruit 48 patients (16/group) to allow for a 10% dropout rate during the study.

### Statistical analysis

Data were presented as mean ± SD (or ± standard error of measurement: SEM) for continuous variables and as absolute frequency and percentage for categorical variables. Baseline differences between groups were tested using univariate ANOVA for continuous variables and the chi-square test for categorical variables. Repeated-measures ANOVA was performed to evaluate differences between the 3 interventions, changes in the outcome variable over time, and whether the changes over time were different between the interventions (interaction term). Post hoc analysis [Fisher's Least Significant Difference (LSD)] was conducted to evaluate significant changes within each intervention after 30 d and 60 d compared with the respective baseline values. The distribution of residuals was checked for normality. ANOVA was used to test differences in changes from baseline between the 3 intervention groups. Post hoc analysis (Fisher's LSD) was conducted to evaluate significant differences for the N + P and N + F interventions compared with the placebo group (P + P). Pearson's correlation analysis was used to evaluate the associations of changes in biomarker concentrations with changes in diastolic and systolic BP. In addition, correlation analysis was performed to evaluate the association between changes in salivary nitrite measured by the salivary strips and chemiluminescence. Statistical analyses were carried out using Statistica 10 for Windows (StatSoft) and a *P* < 0.05 was considered as statistically significant.

## Results

### Recruitment flowchart

**FIGURE 1:**
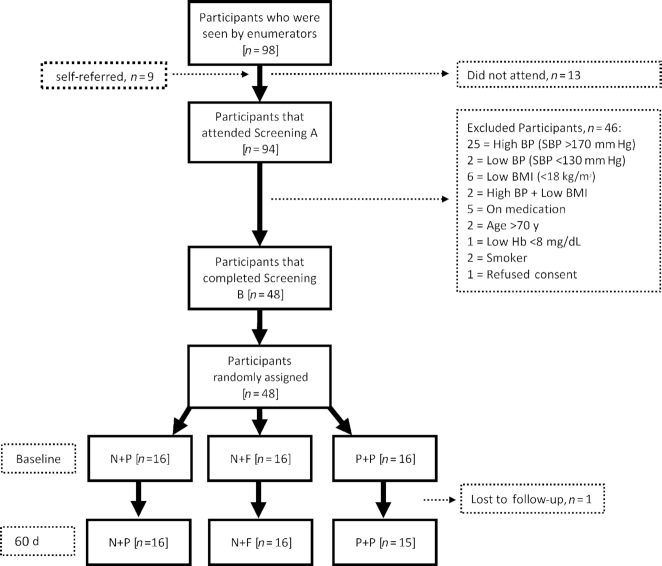
Flowchart describing the number of participants enrolled at each phase of the study from recruitment to completion of the intervention. Screening A was the main part of the screening process as primary measurements were performed to identify eligible participants; the screening B visit was introduced before the baseline visit to ensure commitment to the study, collect qualitative information before the intervention, and conduct a within-subject study to assess the repeatability of clinic and ambulatory BP measurements. BP, blood pressure; F, folic acid; Hb, hemoglobin; N, high-nitrate beetroot juice; P, placebo; SBP, systolic blood pressure.

In total, 94 participants were screened over an 8-wk period between March and April 2018, with baseline visits starting in May 2018. Forty-eight participants met the inclusion criteria and were successfully randomly assigned to 1 of the 3 interventions. Four individuals were unable to complete the prebaseline assessments at screening B. One participant was lost to follow-up because they moved to a new city and so interventions were dispensed to 47 participants at baseline (**Figure 1**).

### Demographics

**Table 1** shows the baseline characteristics of the cohort (for full details see **Supplemental Table 2**). There were 8 (16.7%) men and 40 (83.3%) women with a mean age of 61.8 and 60.0 y, respectively. Forty (83.3%) belonged to the Chagga tribe and 28 (58.3%) members of the cohort had an occupation that involved farming. Active smokers or users of tobacco were excluded. Apart from gender, groups were generally well matched across all variables.

**TABLE 1:** Baseline data of participants enrolled in the study presented for the whole sample and as randomly assigned to N + F, N + P, or P + P interventions^1^

	Total (*n* = 47)	N + P (*n* = 16)	N + F (*n* = 16)	P + P (*n* = 15)	*P* value
Age, y	60.7 ± 6.3	60.0 ± 6.7	61.0 ± 6.6	61.2 ± 5.9	0.85
Gender, % female	83	94	88	67	0.14
Anthropometry					
Height, cm	162.2 ± 8.3	162.4 ± 7.4	161.9 ± 7.9	161.4 ± 9.3	0.98
Weight, kg	72.3 ± 15.3	76.6 ± 15.4	69.0 ± 13.1	71.4 ± 17.3	0.36
Waist circumference, cm	98.3 ± 11.0	99.6 ± 10.0	97.9 ± 11.5	97.2 ± 12.2	0.82
BMI, kg/m^2^	27.6 ± 5.4	29.1 ± 5.8	26.3 ± 4.7	27.3 ± 5.7	0.23
Blood pressure					
CBPM systolic, mm Hg	151.0 ± 19.4	155.8 ± 18.6	150.0 ± 23.9	147.0 ± 14.5	0.45
CBPM diastolic, mm Hg	91.8 ± 11.7	94.0 ± 10.5	92.0 ± 15.0	89.2 ± 9.0	0.52
ABPM systolic,^2^ mm Hg	140.7 ± 14.0	143.7 ± 13.1	139.7 ± 14.0	138.3 ± 11.2	0.55
ABPM diastolic,^2^ mm Hg	87.0 ± 10.2	91.5 ± 11.1	85.3 ± 10.6	83.8 ± 7.0	0.08
Hypertension grading					
Prehypertensive, *n*	8	1	5	2	0.34
Grade 1, *n*	19	6	6	7	
Grade 2, *n*	20	9	5	6	

1 Values are means ± SDs unless otherwise indicated. ABPM, ambulatory blood pressure measurement; CBPM, clinic blood pressure measurement; N + F, high-nitrate beetroot juice and folic acid; N + P, high-nitrate beetroot juice and placebo; P + P, nitrate-depleted beetroot juice and placebo.

2 ABPM *n* = 44 because 3 participants refused to perform the ABPM.

### Baseline data

Table 1 reports the hypertensive grading, which shows that 39 participants had grade 1 or 2 hypertension but with no overall difference in the distribution of hypertension grading between groups (*P* = 0.34). Mean BMI was in the overweight range for all groups (26.3–29.1) with no difference between groups (*P* = 0.23). Similarly, groups did not differ for baseline values of clinic and ambulatory systolic (*P* = 0.45 and *P* = 0.55, respectively) and diastolic (*P* = 0.52 and *P* = 0.08, respectively) BP.

### Safety

The trial was overall well accepted as confirmed by the high compliance and completion rates. About one-third of the patients reported some minor side effects which, as expected, were more frequent in the N + F group (50% of all cases) but not statistically different between groups (*P* = 0.08) (**Supplemental Table 3**). Medication use during the trial was also not significantly different between groups (*P* = 0.24). Five participants were prescribed BP and antiacid medications while on the trial and a sensitivity analysis was conducted by removing these 5 subjects from the analysis.

### Compliance

**FIGURE 2:**
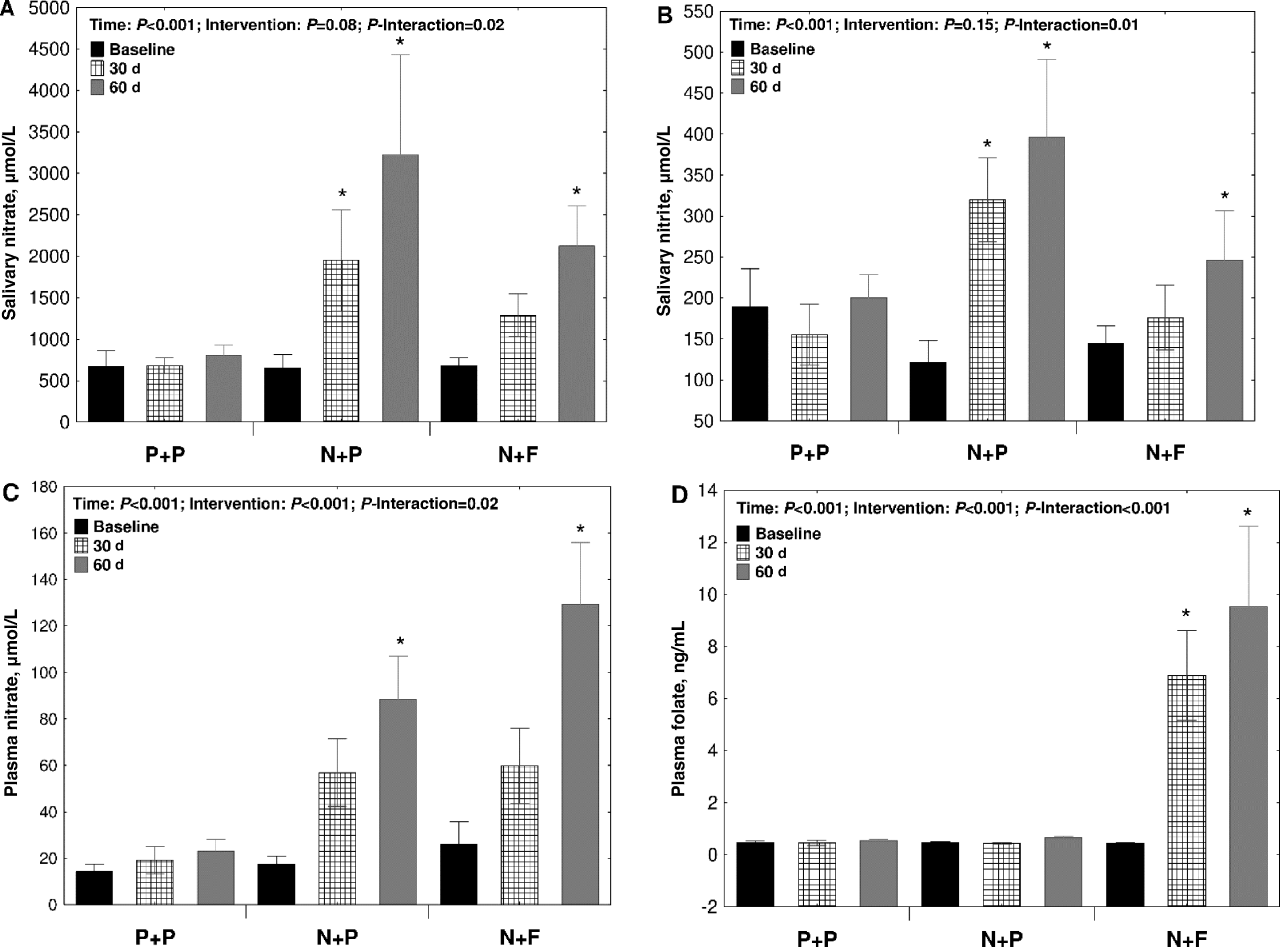
Compliance to the interventions assessed by measurement of salivary nitrate (A) and nitrite concentrations (B) and plasma nitrate (C) and folate concentrations (D) in 47 participants randomly assigned to N + F, N + P, or P + P interventions. Measurements were conducted at baseline, halfway (30 d), and at the end of the study (60 d). *Significant difference compared with baseline within each intervention group (*P* < 0.05). N + F, high-nitrate beetroot juice and folic acid; N + P, high-nitrate beetroot juice and placebo; P + P, nitrate-depleted beetroot juice and placebo. Values are means ± SEs.

All participants reported a high level of compliance to the trial as they declared having taken >90% of the prescribed supplemented products. Gastrointestinal symptoms were the primary reason for participants to skip a daily dose (Supplemental Table 3). The high level of reported compliance is mirrored in the changes in salivary nitrate and nitrite and plasma nitrate and folate concentrations. Salivary nitrate (*P*-interaction = 0.02) (**Figure 2**A) and nitrite (*P*-interaction = 0.01) (Figure 2B) concentrations increased progressively with time in the N + P and N + F groups, whereas the concentrations remained unchanged in the P + P group. Plasma nitrate also increased significantly over time in the nitrate-supplemented groups and the N + F group showed greater changes than the N + P group (*P*-interaction = 0.02) (Figure 2C). As expected, plasma folate only increased in the N + F group (*P*-interaction = 0.02) (Figure 2D). Body weight remained stable during the study, indicating an overall similar energy balance in the 3 groups during the 2-mo intervention (**Supplemental Figure 1**).

This study also offered, to our knowledge for the first time, the opportunity to evaluate the validity of the salivary strips to monitor compliance to high-nitrate prolonged interventions conducted in nonlaboratory settings in developing countries. The study found that the salivary strips were able to detect changes in salivary nitrite concentrations in the 2 nitrate-rich interventions, whereas no changes were observed in the P + P group (**Supplemental Figure 2**A). A significant but modest correlation was also found between changes in scores of the salivary strips and changes in salivary nitrite concentrations (*r* = 0.29, *P* = 0.005) (Supplemental Figure 2B).

### Clinic resting BP

**FIGURE 3:**
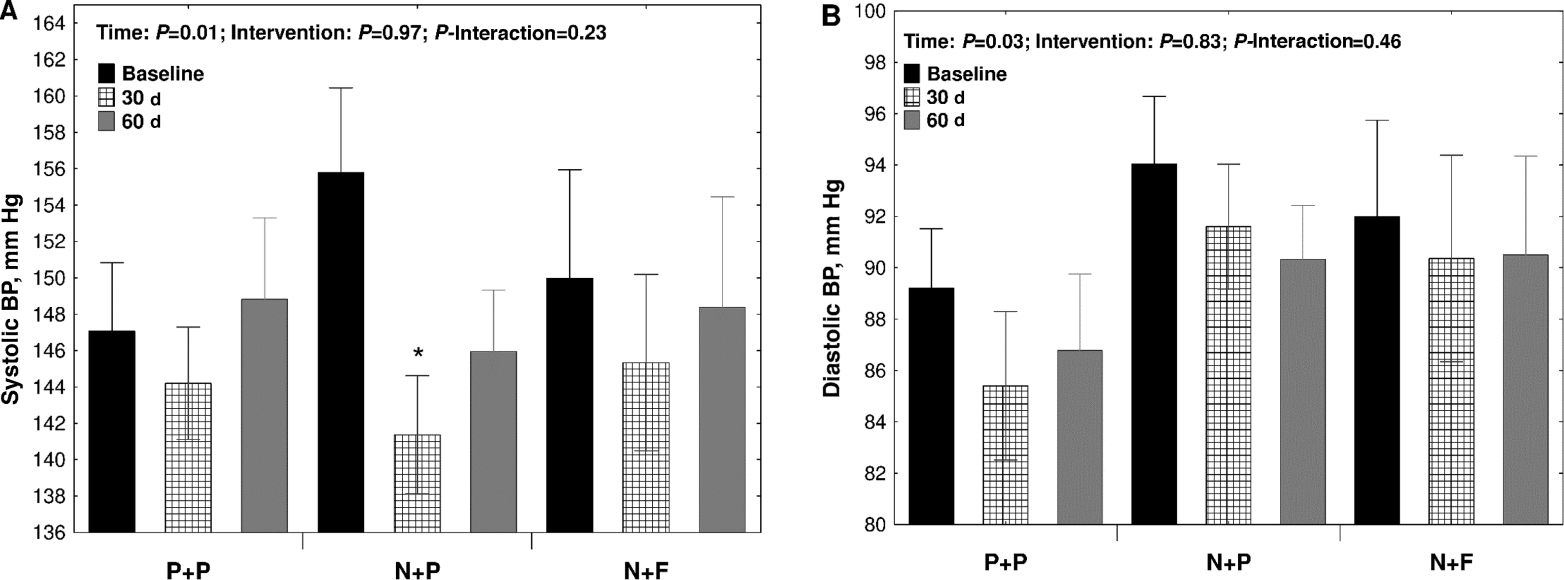
Mean resting clinic systolic (A) and diastolic (B) BP readings in 47 participants randomly assigned to N + F, N + P, or P + P interventions. Measurements were conducted at baseline, halfway (30 d), and at the end of the study (60 d). *Significant difference compared with baseline within each intervention group (*P* < 0.05). Values are means ± SEs. BP, blood pressure; N + F, high-nitrate beetroot juice and folic acid; N + P, high-nitrate beetroot juice and placebo; P + P, nitrate-depleted beetroot juice and placebo.

Resting systolic and diastolic BP did not show significant differences between groups (**Figure 3**A, B). However, the N + P group showed a significant reduction in systolic BP after 30 (−14.4 ± 23.7 mm Hg, *P* = 0.007) and 60 d (−8.8 ± 16.6 mm Hg, *P* = 0.01) compared with baseline (Figure 3A).

### Twenty-four-hour ambulatory BP

**FIGURE 4:**
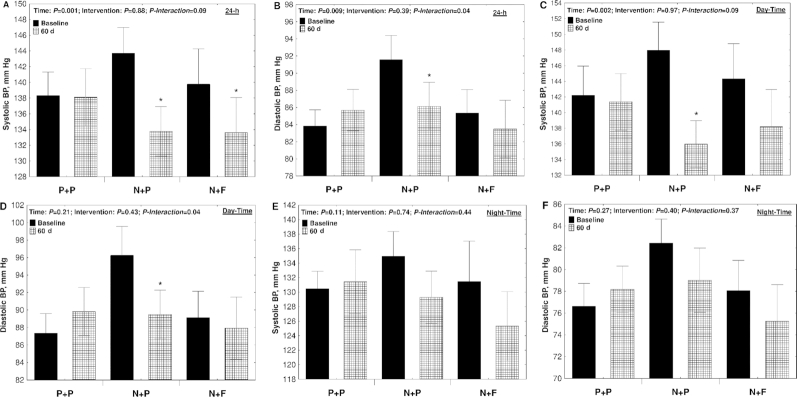
Mean systolic and diastolic 24-h ambulatory BP readings in 44 participants randomly assigned to N + F, N + P, or P + P interventions. Measurements were reported for the 24-h monitoring period (24-h; A and B) and further divided into measurements taken during the day (Day-Time; C and D) and at night (Night-Time; E and F). Twenty-four-hour ambulatory BP measurements were performed at baseline and at the end of the study (60 d). *Significant difference compared with baseline within each intervention group (*P* < 0.05). Values are means ± SEs. BP, blood pressure; N + F, high-nitrate beetroot juice and folic acid; N + P, high-nitrate beetroot juice and placebo; P + P, nitrate-depleted beetroot juice and placebo.

Both N + P and N + F interventions had a significant effect (time, *P* = 0.001; intervention, *P* = 0.88; *P*-interaction = 0.08) on 24-h systolic BP. After 60 d, 24-h systolic BP dropped by −10.8 ± 9.8 mm Hg (*P* < 0.001) and −6.1 ± 13.2 mm Hg (*P* = 0.03) in the N + P and N + F groups, respectively (**Figure 4**A). Twenty-four-hour diastolic BP only decreased significantly in the N + P group (−5.4 ± 5.0 mm Hg, *P* = 0.004) (Figure 4B). The decrease in day-time systolic and diastolic BP was significant only for the N + P group (−11.9 ± 11.0 mm Hg, *P* = 0.001 and −6.7 ± 8.6 mm Hg, *P* = 0.006, respectively) (Figure 4C, D). No significant effects were observed on night-time systolic and diastolic BP (Figure 4E, F).

### Sensitivity analyses

The percentage of valid 24-h BP recordings at baseline and end of the study was ∼59% ± 17%. Thirty-three subjects had valid 24-h BP recordings >50% at both baseline and end of the study and a significant drop in both 24-h systolic and diastolic BP was still observed when only these subjects were included in the analysis (**Supplemental Table 4**). In addition, the exclusion from the analysis of 5 subjects taking antihypertensive and antiacid medications during the trial did not affect the results because a significant drop was observed in 24-h systolic and diastolic BP in the N + P group (**Supplemental Table 5**).

### Biomarkers

Plasma homocysteine, nitro-tyrosine, and CRP concentrations were measured at baseline and after 30 and 60 d. Plasma homocysteine showed a tendency to increase in the placebo group and a significant decrease was seen after 60 d in the N + F group (*P* = 0.05) (**Supplemental Figure 3**A). No significant changes were observed during the trial for plasma nitro-tyrosine (*P*-interaction = 0.67) (Supplemental Figure 3B) and CRP (*P*-interaction = 0.54) (Supplemental Figure 3C) concentrations.

## Discussion

The principal original finding of this study is that dietary supplementation with nitrate-rich beetroot juice, alone or in combination with folic acid, results in clinically significant reductions in BP in drug-naïve Tanzanian adults. These findings have important implications for the treatment of hypertension and prevention of cardiovascular diseases (CVDs) in Sub-Saharan Africa.

The effects of dietary inorganic nitrate supplementation on BP have been investigated in previous studies, with a recent meta-analysis reporting overall reductions in both systolic (∆ 4.8 mm Hg) and diastolic (∆ 1.7 mm Hg) BP consequent to nitrate consumption (13). The majority of previous investigations have explored the effects of acute or short-term nitrate ingestion on BP, were conducted in high-income countries (typically in the United States, United Kingdom, or Sweden), and examined the effects of nitrate alone, without exploring possible additive or synergistic effects with other compounds. Our study is unique, because of the prolonged (60-d) supplementation period, Sub-Saharan African setting, and inclusion of a nitrate plus folic acid experimental arm. We found that nitrate supplementation alone elevated markers of NO bioavailability, reduced clinic and ambulatory systolic BP by ∼10 mm Hg, and lowered diastolic ambulatory BP by ∼7 mm Hg. These BP reductions are similar in magnitude to those reported by Webb et al. (12) after acute nitrate-rich beetroot juice ingestion (resting clinic BP: ∼10/8 mm Hg reduction) and Kapil et al. (22) after 4 wk of nitrate-rich beetroot consumption (ambulatory BP: ∼8/5 mm Hg reduction). To contextualize our findings, a meta-analysis (23) demonstrated that a 10 mm Hg reduction in systolic BP would be expected to reduce the risk of major cardiovascular events by 20%, coronary artery disease by 17%, stroke by 27%, heart failure by 28%, and all-cause mortality by 13%. Because participants exhibited relatively few side effects to nitrate consumption, and compliance was good, this suggests that nitrate could represent an effective nutritional agent to reduce BP and improve cardiovascular health in Tanzania.

Inorganic nitrate and folic acid have been proposed to reduce BP via different mechanisms (13, 16–18). Therefore, we reasoned that combined supplementation with these 2 compounds could elicit greater BP-reducing effects than nitrate alone. Although we found similar effects of nitrate plus folic acid compared with nitrate alone on systolic ambulatory BP (∼7 compared with ∼10 mm Hg), combined supplementation with these 2 compounds had no effects on diastolic ambulatory BP or clinic-based BP. Tentatively, this may indicate that the BP-lowering effects of nitrate are partly diminished (but still clinically relevant) when this inorganic anion is ingested alongside folic acid, although further research is needed to confirm this hypothesis and to investigate potential mechanisms. Interestingly, previous studies have indicated that the wider dietary context can modulate the effects of inorganic nitrate on BP. For example, 1 study found that the antihypertensive effects of nitrate were abolished when this compound was consumed alongside thiocyanate-rich vegetables such as broccoli (24), whereas another suggested potential BP-increasing effects of nitrate when consumed alongside dietary sulfate (25).

Conversely, we recently found that the BP-lowering effects of nitrate supplementation were enhanced with coadministration of vitamin C (26). Understanding the interactions between nitrate and other dietary components represents an important area of exploration for future studies. It is noteworthy that plasma homocysteine tended to be lower in the nitrate plus folic acid condition in our study—an effect previously well established with folic acid consumption (27). Because elevated homocysteine concentrations have been associated with increased risk of CVD, this could represent a BP-independent mechanism through which nitrate and folate combined could reduce CVD risk. Longer-duration studies exploring the effects of nitrate alone, or in combination with folate, on BP and hard clinical endpoints (e.g., CVD incidence and mortality) would be valuable in the future.

Our results provide proof-of-concept that dietary nitrate supplementation may represent an effective way of reducing BP in hypertensive Tanzanian individuals—effects that were verified both in a controlled clinical environment and in a free-living setting via 24-h ambulatory monitoring. A key question to address going forward is whether similar benefits can be achieved through consumption of whole foods such as nitrate-rich salads which can be grown locally in Tanzania and produced at a low cost, rather than having to rely upon commercial nutritional supplements such as the highly concentrated beetroot juice used in this study. Blekkenhorst et al. (28) recently explored the efficacy of a nitrate-based whole-food intervention on BP in a group of Australian individuals with mildly elevated BP. Those authors observed excellent compliance (∼98%) to a 4-wk intervention with nitrate-rich vegetables, which resulted in a mean increase in nitrate intake of ∼150 mg/d. However, BP was not reduced, which may be due to the relatively modest amount of nitrate consumed, background diet, participants’ characteristics, or other methodological factors. Ensuring a sufficiently high nitrate dose verified before intervention in an “at-risk” population with raised BP may help maximize the chance of observing a beneficial effect of nitrate-rich foods on BP in future studies (29). Further research into the long-term compliance to a nitrate-based intervention is also warranted. In this regard, we showed here potential utility of salivary strips for verifying nitrate consumption in a free-living setting, which substantiates previous laboratory research (30).

In conclusion, the present study provides preliminary evidence that dietary inorganic nitrate supplementation, alone or in combination with folic acid, represents a potential nutritional strategy to help combat the burgeoning hypertension epidemic in Tanzania and other Sub-Saharan African countries. These findings support the rationale for future long-term investigations exploring the efficacy of dietary nitrate for reducing BP and attenuating CVD risk in this setting and other LMIC settings.

## Supplementary Material

nxaa170_Supplemental_File

## Notes

Supported by Medical Research Council Newcastle Confidence in Concept grant BH171899 (to RW).

Author disclosures: The authors report no conflicts of interest.

The funders had no role in the study design, data collection, analysis and interpretation, the preparation of the manuscript, or the decision to submit the manuscript for publication.

Supplemental Tables 1–5, Supplemental Figures 1–3, and Supplemental Methods are available from the “Supplementary data” link in the online posting of the article and from the same link in the online table of contents at https://academic.oup.com/jn/.

The data sets used and/or analyzed during the current study are available from the corresponding author on reasonable request.

MS and OS contributed equally to this work as first authors. BTM and RW contributed equally to this work as last authors.

Abbreviations used: ABPM, ambulatory 24-h blood pressure monitoring; BP, blood pressure; CBPM, clinic blood pressure measurement; CRP, C-reactive protein; CVD, cardiovascular disease; KCRI, Kilimanjaro Clinical Research Institute; LMIC, low- and middle-income country; LSD, Least Significant Difference; N + F, high-nitrate beetroot juice and folic acid; N + P, high-nitrate beetroot juice and placebo; P + P, nitrate-depleted beetroot juice and placebo.
