# Supplementary material for: Nitrate-Rich Beetroot Juice Reduces Blood Pressure in Tanzanian Adults with Elevated Blood Pressure: A Double-Blind Randomized Controlled Feasibility Trial
Source: J Nutr. 2020 Jul 29;150(9):2460–8. doi: 10.1093/jn/nxaa170 (PMC7467850; doi:10.1093/jn/nxaa170)
Supplement: nxaa170_Supplemental_File [file nxaa170_supplemental_file.docx]

**Nitrate-rich beetroot juice reduces blood pressure in Tanzanian adults with elevated blood pressure: A double-blind randomized controlled feasibility trial**

Mario Siervo et al.

**Online Supplementary Material**

| Supplemental Table 1: Exclusion Criteria  • Smoking  • Ongoing participation in other clinical studies  • Physical disabilities that limit mobility  • Vegetarian diet (would have high nitrate intake)  • Inability to comply with study diet  • Significant weight change in the previous 3 months (> 5 kg)  • Active cancer or previous diagnosis of malignant cancer within the last 5 years  • Chronic or acute metabolic and inflammatory conditions (Rheumatoid Arthritis, inflammatory bowel diseases, severe liver disease)  • Type 1 or type 2 Diabetes treated with Insulin  • On medication (Diuretics, Oral corticosteroids, Laxatives, Anticoagulants, Nitrate derived agents, Anti-cholinergics and Anti-hypertensives such as Ace-inhibitors, Angiotensin receptor inhibitors, Beta blockers or Calcium channel antagonists)  • History of severe anemia (Hb < 8mg/dL)  • Current diagnosis of severe infectious diseases (e.g HIV, malaria, hepatitis, yellow fever)  • History of major surgical operations.  • Participants taking hormonal therapies (estrogens, thyroxine, progesterone, oral hypoglycemic agents), antidyslipidemic (statins) and psychiatric drugs (antidepressants, sedatives, antipsychotics) were excluded if they had either started or altered their dose within the last three months. |
| --- |

| Supplemental Table 2: Additional data on baseline characteristics of the participants collected at screening visit B and presented for the whole sample (n=48) and for each intervention group.^1^ | | | | | |
| --- | --- | --- | --- | --- | --- |
|  |  | Total (n=48) | N+P  (n=16) | N+F  (n=16) | P+P  (n=16) |
| *Religion (%)*  Islam  Christian  No data | | 58.3  35.4  6.3 | 68.8  25.0  6.3 | 62.5  37.5  0.0 | 43.8  43.8  12.5 |
| *Tribe (%)*  Chagga  Maasai  Haya  Nyaturu  Pare | | 83.3  2.1  4.2  2.1  8.3 | 75.0  0.0  6.3  6.3  12.5 | 93.8  6.3  0.0  0.0  0.0 | 81.3  0.0  6.3  0.0  12.5 |
| *Marital status (%)*  Married  Widowed  Divorced  No data | | 56.3  27.1  10.4  6.3 | 50.0  37.5  6.3  6.3 | 62.5  12.5  25.0  0.0 | 56.3  31.3  0.0  12.5 |
| *Occupation (%)*  Farming/Animal Keeping  Home-keeping  Farming and Trading  Trading  Unemployed  Skilled profession*  Missing Data | | 45.8  20.8  12.5  8.3  4.2  4.2  4.2 | 37.5  25.0  12.5  6.3  6.3  6.3  6.3 | 56.3  12.5  6.3  18.8  6.3  0.0  0.0 | 43.8  25  18.8  0.0  0.0  6.3  6.3 |
| *Alcohol (%)*  Never  Ex-drinker  Currently Drinking | | 39.6  25.0  35.4 | 37.5  18.8  43.8 | 37.5  25.0  37.5 | 43.8  31.3  25.0 |
| *Smoking (%)*  Never  Ex-smoker | | 75.0  25.0 | 75.0  25.0 | 75.0  25.0 | 75.0  25.0 |
| *Medication Use (n)*  Paracetamol  Metformin  Chlorpropamide  Cetirizine  Doxyciclin  Maloxicen | | 4  1  1  2  1  1 | 1  1  1 | 1  1  1  1 | 2  1 |

^1^*1=Chef, 1 = Car mechanic, n= number of participants. N: High-Nitrate Beetroot Juice; F: Folic Acid; P: Placebo.





Supplemental Figure 1: Mean body weight in 47 participants randomized to high-nitrate beetroot juice and folic acid (N+F), high-nitrate beetroot juice and placebo (N+P) or nitrate-depleted beetroot juice and placebo (P+P) interventions. Measurements were conducted at baseline, half-way (30 days) and at the end of the study (60 days). Values are means ± SE.

| Supplemental Table 3: Number of side effects and medication use reported by participants enrolled in the study and presented for each intervention group^1^ | | | | |
| --- | --- | --- | --- | --- |
|  | P+P | N+P | N+F | *P* value |
| Side Effects, n  Tiredness  GI Problems  MSK Pain  Infection  Headache  CV Symptoms  Total | 1  3  1  1  1  1  8 | 3  2  1  2  1  9 | 2  7  4  1  2  1  17 | 0.08 |
| Medications, n  Pain Killers  Antiacids  Antihypertensives  Antibiotics  Total | 6  2  8 | 2  1  1  1  5 | 4  1  2  7 | 0.24 |

^1^Gastrointestinal (GI) effects include diarrhoea, nausea, heartburn, vomiting. Musculoskeletal (MSK). Cardiovascular (CV) effects include palpitations and raised blood pressure. n= number of events. Please note that different events may have been reported by the same individual. N: High-Nitrate Beetroot Juice; F: Folic Acid; P: Placebo.


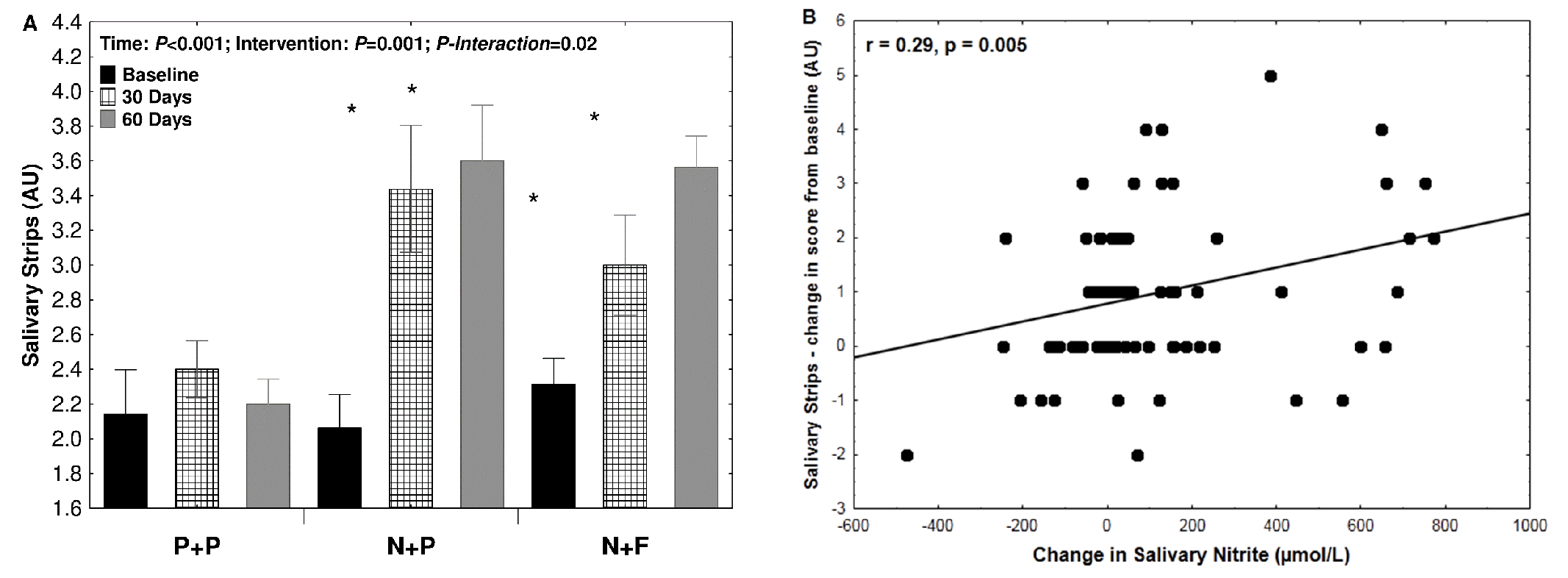


Supplemental Figure 2: Compliance to the interventions assessed by qualitative measurement of salivary nitrite concentrations using salivary strips (see methods for more details) (A) in 47 participants randomized to high-nitrate beetroot juice and folic acid (N+F), high-nitrate beetroot juice and placebo (N+P) or nitrate-depleted beetroot juice and placebo (P+P) interventions. Measurements were conducted at baseline, half-way (30 days) and at the end of the study (60 days). Salivary strips changes in salivary nitrite concentrations, expressed in arbitrary units (AU), from baseline measured at 30 days and 60 days were correlated with changes in salivary nitrite concentrations, measured by ozone-based chemiluminescence, to evaluate the agreement between the two methods (B). *Significant difference compared to baseline within each intervention group (*P* < 0.05). Values are means ± SE.

| Supplemental Table 4: Sensitivity analysis to evaluate whether differences in blood pressure (BP) responses to the interventions were different in participants with more than 50% valid recordings during the 24-hour BP monitoring period at baseline and at the end of the study^1^ | | | |
| --- | --- | --- | --- |
|  | n | Δ | *P* value |
| Systolic BP, mmHg  P+P  N+P  N+F | 10  12  11 | -1.0±9.5  -11.2±10.3*  -6.2±14.8 | 0.31 |
| Diastolic BP, mmHg  P+P  N+P  N+F | 10  12  11 | 1.5±8.2  -4.9±5.6*  -1.8±9.0 | 0.34 |

^1^n= number of participants. Δ= changes in BP from baseline. *Significant difference compared to placebo group (P+P) (P < 0.05). N: High-Nitrate Beetroot Juice; F: Folic Acid; P: Placebo. Values are means ± SDs.

| Supplemental Table 5: Sensitivity analysis to evaluate whether differences in blood pressure (BP) responses to the interventions were different after exclusion (n=5) of participants taking anti-hypertensive and anti-acid medications during the study^1^ | | | |
| --- | --- | --- | --- |
|  | n | Δ | *P* value |
| Systolic BP, mmHg  P+P  N+P  N+F | 10  14  15 | -0.3±9.7  -10.8±13.2*  -6.1±9.8 | 0.27 |
| Diastolic BP, mmHg  P+P  N+P  N+F | 10  14  15 | 1.3±9.1  -6.2±4.9*  -1.8±8.1 | 0.21 |

^1^n= number of participants. Δ= changes in BP from baseline. *Significant difference compared to placebo group (P+P) (P < 0.05). N: High-Nitrate Beetroot Juice; F: Folic Acid; P: Placebo. Values are means ± SDs.

**

**

Supplemental Figure 3: Measurement of homocysteine (A), nitro-tyrosine (B) and C reactive protein (C) concentrations in 47 participants randomized to high-nitrate beetroot juice and folic acid (N+F), high-nitrate beetroot juice and placebo (N+P) or nitrate-depleted beetroot juice and placebo (P+P) interventions. Measurements were conducted at baseline, half-way (30 days) and at the end of the study (60 days). *Significant difference compared to baseline within each intervention group (*P* < 0.05). Values are means ± SE.

**Supplemental methods**

Ozone-based chemiluminescence

Plasma samples were first deproteinised before analysis using cold ethanol precipitation. The required ethanol was first chilled to 0°C. 0.5ml of each sample was placed in a 1.5ml microcentrifuge tube and 1ml of cold ethanol was added. The tubes were then vortexed for 10 seconds and placed at 0°C for 30 minutes. Samples were centrifuged at 15,000 x g for 5 minutes. The supernatant was removed for the determination of nitrate. Saliva samples were diluted 1:100 prior to analysis. The ozone-based chemiluminescence method was used to measure plasma and salivary nitrate and nitrite and urinary nitrate concentrations using the Sievers gas-phase chemiluminescence nitric oxide analyser (NOA 280i, Analytix, UK). Briefly, prior to analysis a standard curve was created for both nitrate and nitrite and an equation was derived to calculate nitrate and nitrite concentrations by fitting a linear regression line to the standard concentrations. R^2^ of the regression lines was greater than 0.99. Diluted samples were injected into the purge vessel using a glass Hamilton (Fisher) syringe with an injection volume ranging from 10µL to 100µL. The injection volume was adjusted to maximise the sensitivity of the measurements and taken into account into the calculations of the final concentrations. The area of the curve of the peaks was calculated automatically by the Analytix software and used to calculate the concentrations of the samples using the standard regression equations. Samples were analysed in singlicate and quality of the peaks was checked visually based on height, fronting and tailing. Analyses were repeated for samples with low quality peaks. The assay performance of the nitrate and nitrite analysis is very high. We found a within-operator CV% of 91% for nitrate and 89% for nitrite after a series of ten consecutive manual injections of a standard solution with known concentration. The calculations were based on the AUCs calculated automatically for each peak by the software which minimise the risk of between-operator differences in the calculation of the results.

**Supplemental references**

1. Whitehead AL, Julious SA, Cooper CL, Campbell MJ. Estimating the sample size for a pilot randomised trial to minimise the overall trial sample size for the external pilot and main trial for a continuous outcome variable. Statistical Methods in Medical Research. 2016;25(3):1057-73. doi:10.1177/0962280215588241

2. Kapil V, Khambata RS, Robertson A, Caulfield MJ, Ahluwalia A. Dietary nitrate provides sustained blood pressure lowering in hypertensive patients: a randomized, phase 2, double-blind, placebo-controlled study. Hypertension. 2015;65(2):320-7. doi:10.1161/HYPERTENSIONAHA.114.04675
